# Codetection of *Trichomonas vaginalis* and *Candida albicans* by PCR in Urine Samples in a Low-Risk Population Attended in a Clinic First Level in Central Veracruz, Mexico

**DOI:** 10.1155/2013/281892

**Published:** 2013-08-29

**Authors:** A. López-Monteon, F. S. Gómez-Figueroa, G. Ramos-Poceros, D. Guzmán-Gómez, A. Ramos-Ligonio

**Affiliations:** ^1^LADISER Inmunología y Biología Molecular, Facultad de Ciencias Químicas, Universidad Veracruzana, 94340 Orizaba, VER, Mexico; ^2^Centro de Investigaciones Biomédicas, Universidad Veracruzana, 91000 Xalapa, VER, Mexico

## Abstract

The aim of this study is to estimate the prevalence of *Trichomonas vaginalis* and *Candida albicans* in low-risk patients treated at a first level clinic (primary health care represents the first level of contact of individuals, families, and the community with the system national health). Using a cross-sectional study in patients treated in clinical laboratory of the Sanitary District no. 7 of the city of Orizaba during the months June-July, 252 urine samples were collected for the identification of *T. vaginalis* and *C. albicans* by PCR. Furthermore, we analyzed the sociodemographic characteristics of the studied population. We observed an overall prevalence of 23.41% (95% CI 22.10–24.72) for *T. vaginalis* and 38.88% (95% CI 37.73–40.03) for *C. albicans*. There was also presence of coinfection in 14.28% (95% CI 13.10–15.46), which was associated with the presence of pain. Most of the positive cases were observed in women house-maker (80%, 95% CI 50.36–48.98). The results of this study provide evidence that the majority of positive cases observed in the studied population are presented in an asymptomatic form and usually are not associated with any risk factor.

## 1. Introduction

Sexually transmitted infections (STIs) are the second leading cause of morbidity in young women in developing countries, after causes related to pregnancy and childbirth [1]. It is estimated that the number of people suffering from curable STIs in the world per year is approximately 340 million. Among the STIs considered curable by World Health Organization (WHO) [2] stand out those caused by etiological agents *Neisseria gonorrhoeae*, *Chlamydia trachomatis*, and *Trichomonas vaginalis* [3]. The impact of these STIs as a public health problem occurs not only for its high prevalence, but also due to the evolution of these infections in an acute phase and in a chronic phase with sequelae due to a lack of accurate diagnosis and appropriate treatment: the pelvic inflammatory processes, perinatal morbidity, and infertility [1]. *T. vaginalis* is a flagellated, parasitic protozoan, which causes trichomoniasis by infecting urogenital tract. Trichomoniasis is one of the most common causes of nonviral genitourinary sexually transmitted infection (STI) in humans, with a worldwide prevalence of 174 million cases annually. According to WHO estimation, it accounts for almost half of all curable STIs [3]. In spite of high prevalence, it is one of the poorly studied parasites with respect to virulence properties, pathogenesis, and immunopathogenesis. *T. vaginalis* infection is asymptomatic in about 50% of infected women and in over 90% of men; thus, re-infection and reexposure is problematic. Furthermore, coinfections among these three STIs are common [4].

*C. albicans* is a dimorphic fungus that colonizes different areas of the body from the gastrointestinal tract to oral and vaginal mucosa. It is usually a commensal microorganism but in immunocompromised or otherwise debilitated hosts it can cause disseminated and mucosal candidiasis [5]. *Candida* species are the second most common cause of vulvovaginitis worldwide. The prevalence of vulvovaginal candidiasis (VVC) is increasing due to the extensive utilization of broad-spectrum antibiotics as well as increased cases of immunocompromised patients; *C. albicans* is the most common and clinically relevant species, that is, present in 85–90% of VVC [6]. However, there has been a significant trend towards the emergence of other species, which ironically show more resistance to the first line antifungal treatments [7]. As *Trichomonas *prevalence depends on factors such as age, number of sexual partners, and pattern of sexual activity, a better understanding of the local epidemiology of the infection in women would be useful in planning prevention strategies [8]. In 2010, 124,310 cases for *T. vaginalis* were reported in Mexico and 293,530 candidiasis cases, with a higher incidence in 25- to 44-year-old persons and a national incidence rate of 114.68/100,000 and 270.79/100,000 in habitants, respectively, where the state of Veracruz was ranked in first place in the number of cases for both infections, with an incidence rate of 224.5/100,000 for trichomoniasis and 480.4/100,000 for candidiasis [9]. Shortages of basic data on the true incidence and prevalence of STIs do not allow reliable information to estimate the impact of the transmission of these diseases. The aim of this study was to estimate by PCR the prevalence of *T. vaginalis* and *C. albicans *and to describe trends of positiveness in patients attending at a first level clinic in Orizaba, Veracruz, Mexico.

## 2. Materials and Methods

### 2.1. Study

**Figure 1:**
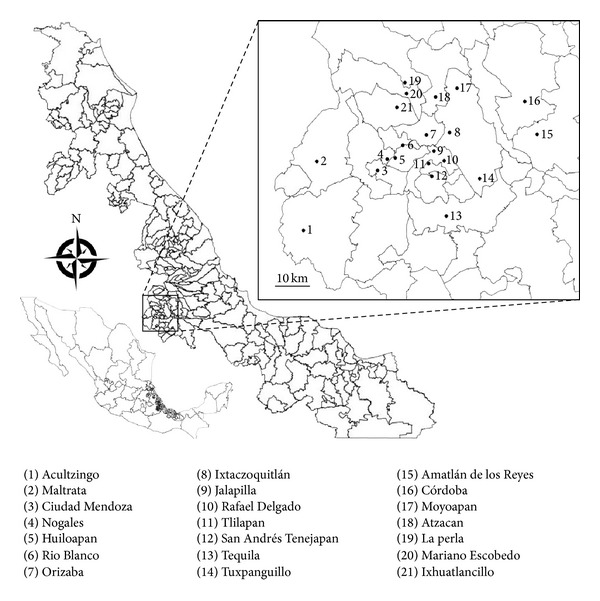
Mexico (bottom left), the state of Veracruz (center), and the study area (inset). Lines delimitating the respective municipalities. (1)–(21), indicate the name of the villages in the different municipalities.

The cross-sectional study was conducted on patients, who received attention in the clinical laboratories of the health jurisdiction VII from Orizaba, Veracruz, between June 19, 2012 and July 10, 2012. Those who provided written informed consent were enrolled in this study. Patients treated at this clinic come from various locations all belonging to the state of Veracruz (Figure 1). Sociodemographic characteristics and gynecological complaints were obtained in private using structured questionnaires applied by one investigator. Criteria for inclusion in this study were belonging to the clinic population, being within the mentioned age group, and voluntariness of the patient. Exclusion criteria were patient refusal and inability to give informed consent. All participants provided a sample of 10 mL urine collected in the morning. The samples were received in the laboratory of the sanitary jurisdiction and stored at −20°C and transported to LADISER Inmunología y Biología Molecular for further processing. 

### 2.2. Obtaining DNA of Urine Samples

252 urine samples were collected. The patient was also asked to provide 10 mL of urine which was pelleted in its entirety at 2,000 ×g for 10 min, the supernatant was removed, and the pellet was rinsed in 1 mL of phosphate buffered saline (PBS (137 mM NaCl, 2.7 mM KCl, 4.3 mM Na_2_HPO_4_, 1.4 mM KH_2_PO_4_, pH 7.4)) and repelleted at 2,000 ×g for 10 min. The supernatant was discarded, and the pellet was frozen at −20°C. DNA was extracted as previously described with some modification [10]. Briefly, thawed samples were resuspended in 600 *μ*L of lysis buffer (1 M Tris, 0.5 M EDTA, 10% glucose, and lysozyme 2 mg/mL), heated at 80°C for 5 min, and then cooled to room temperature. The samples were RNase treated (Promega, Madison, WI, USA) (0.5 mg/mL) for 1 h at 37°C. Proteins were precipitated with 0.2 N NaOH, 1% sodium dodecyl sulfate, 5 M CH_3_COOK (pH 4.8) for 5 min on ice and then centrifuged for 3 min at 2,000 ×g. DNA was precipitated with 600 *μ*L of isopropanol and then centrifuged for 3 min at 2,000 ×g, and then the DNA pellet was washed with 600 *μ*L of 70% ethanol and centrifuged for 3 min at 2,000 ×g. The DNA pellet was dried, resuspended in 50 *μ*L of 10 mM Tris (pH 7.4), 1 mM EDTA (pH 8.0), and heated at 65°C for 1 h. The presence of genomic DNA was confirmed in each sample by electrophoresis prior to PCR amplification. 

### 2.3. PCR for *T. vaginalis* and *C. albicans*

*T. vaginalis*-specific primers TV3 (5′-ATT GTC GAA CAT TGG TCT TAC CCT C-3′) and TV7 (5′-TCT GTG CCG TCT TCA AGT ATG C-3′) [11] and *C. albicans*-specific primers Calb-1 (5′-AAG TAT TTG GGA GAA GGG AAA GGG-3′) and Calb-2 (5′-AAA ATG GGC ATT AAG GAA AAG AGC-3′) [12] were used for PCR amplification. The PCR mixture consisted of 5 *μ*L of 10 x PCR buffer, 4 *μ*L of deoxynucleoside triphosphates (2.5 mM each), 0.5 *μ*L of each primer pair (10 pmol/*μ*L), 0.5 *μ*L of Taq DNA polymerase (Promega) (5 U/mL), 10 *μ*L of sample (5 to 10 ng/mL), and 29.5 *μ*L of distilled water. Positive and negative controls were included in all PCR runs. The positive control consisted of DNA from ATCC *T. vaginalis* isolate 30184 and ATCC *C. albicans* isolate 18804. Negative controls included DNA from *T. cruzi* MHOM/MX/1994/INC-1 strain, PCR mix with primers but no DNA, and human genomic DNA. PCR amplification consisted of 30 cycles of 1 min at 90°C, 30 s at 60°C, and 2 min at 72°C for *T. vaginalis*, and 40 cycles of 60 s at 94°C, 30 s at 55°C, and 45 s at 62°C for *C. albicans*. After amplification, there was an additional extension step at 72°C for 7 min, and then the samples were cooled to 4°C. Five microliters of amplified product was electrophoresed on a 1.8% agarose, 0.5 mg/mL ethidium bromide gel, viewed on a UV light box, and photographed. Samples containing a 300 bp fragment were considered positive for *T. vaginalis, *and samples containing a 310 bp fragment were considered positive for *C. albicans.*

### 2.4. Statistical Methods

Frequency distribution of demographic data, characteristics of the population, sexual history, and clinical manifestations were analyzed. The relationship between selected risk factors and the prevalence of trichomoniasis and candidiasis were compared using *χ* ^2^ or Fisher's exact test when appropriate. Ninety-five percent confidence intervals were calculated to evaluate statistically significant differences between collection methods. The relationship between age and seroprevalence rate was assessed by chi-square test and by regression analysis. All municipalities were referenced and the data were entered into a geographical information system database in Qgis version 1.8 to generate maps.

## 3. Results

The 252 samples were divided into 7 age groups (Table 1), age of the participants ranged from 14 to 90 years old, the group between 21 and 30 years had more number of samples: 46 samples (18.25%); education level showed that from the total studied population, 34.52% (CI 95% 33.01–36.03) have elementary school, 27.77% (CI 95% 25.97–29.57) have secondary education, 20.23% (CI 95% 18.31–22.18) have high school education, 4.36% (CI 95% 3.05–5.67) have university studies, and 0.39% (CI 95% −0.35–1.09) have postgraduate studies. In relation to marital status, 33.73% (CI 95% 32.82–34.64) declared to remain single, 35.31% (CI 95% 34.02–36.6) reported being married, and 30.95% (CI 95% 29.86–32.04) said that they are living together, being divorced, or widowed.

In order to establish a history about the presence of STIs in this study group, they were questioned whether they had ever suffered at least one STI in their lives; 96.42% (CI 95% 73.81–119.02) reported not having had an STI; the remaining 9 participants (3.57%, CI 95% 2.76–4.38) agreed to have suffered at least one STI but did not reveal the causal agent of it. Similarly, they were asked whether they regularly used condoms in their sexual relations; 5.55% (9/252, CI 95% 4.51–6.59) have indicated consistent condom use. In relation to smoking and consumption of alcohol, 3.17% (8/252, CI 95% 2.24–4.10) of the studied population is smokers, and 99/252 of them (39.28%, CI 95% 38.23–40.33) consume alcohol. 

Finally, in relation to the number of sexual partners, 50.39% (127/252, CI 95% 49.45–51.33) mentioned to have only one partner, 21.42% (54/252, CI 95% 20.38–22.46) mentioned to have two sexual partners, 23.01% (58/252, CI 95% 22.73–23.29) mentioned to have more than two sexual partners, and 0.79% (2/252, CI 95% 0.12–1.46) did not mention sexual partners. 18.65% (47/252, CI 95% 17.87–19.43) mentioned a family history of cancer, and only 23.41% (59/252, CI 95% 22.22–24.60) of the patients have expressed discomfort (burning, itching, and vaginal secretion).

All 252 samples were used to identify the presence of *T. vaginalis* and *C. albicans* by PCR; molecular diagnosis of *T. vaginalis* showed an overall prevalence of 23.41% (59/252, CI 95% 22.10–24.72) (Table 2); all positive samples showed an amplification of 300 bp, finding a mean age of 43.3 years for patients positive for *T. vaginalis*. When we analyzed the presence of *C. albicans* genomic material in the 252 studied samples, 38.88% (98/252, CI 95% 37.73–40.03) (Table 2) samples resulted positive for amplification of 310 bp fragment specific for *C. albicans*, with an average age of 41.9 years, for patients who tested positive for the presence of *C. albicans*. When we analyzed the presence of coinfections, 14.28% (36/252, CI 95% 13.10–15.46) of the samples resulted positive, where 11.11% (28/252, CI 95% 10.16–12.06) turned out to be women devoted to housework and 3.17% (8/252, CI 95% 2.01–4.33) of co-infections were found in samples from men. The presence of co-infection (18/82) was associated with the presence of pain (32.5% versus 67.4%) (*χ* ^2^ = 4.942, degrees  of  freedom = 2, *P* = 0.026 with Yate's corrections). Moreover, of the total number of samples analyzed, 83.3% (210/252) correspond to samples of women, from which 80% (168/210, CI 95% 79.30–80.69) have as occupation housework. Surprisingly, in this group there are 41 samples of the 59 cases positive for *T. vaginalis* and 62 of the 98 cases positive for *C. albicans*, being a high percentage of the total positive samples, 16.26% (41/252, CI 95% 15.18–17.34) and 24.6% (62/252, CI 95% 23.77–25.43) for *T. vaginalis* and *C. albicans*, respectively, in an age range between 51 and 60 years, while 2.77% (7/252) of *T*.* vaginalis* positive cases and 6.34% (16/252) of the cases for *C. albicans* from the women group who are predominantly students in an age range of 14 to 20 years. However, during the association analysis between the occupation and the presence of infection, there existed no significant correlation with infection with *T. vaginalis* (*P* = 0.410, by Fisher's exact test) or infection with *C. albicans* (*P* = 1.0, by Fisher's exact test). It is noteworthy that positive male samples for *T. vaginalis* (11/59), representing 4.3% (11/252, 95% CI 3.14–5.58) of the total studied population, are at an age range above 42 years. Of the total female population (210/252), the 35.71% (75/210, CI 95% 32.24–39.07) were pregnant, of which 7.14% (15/210, CI 95% 5.79–8.49) were positive to *T. vaginalis*, 10.95% (23/210, CI 95% 9.06–12.84) were positive to *C. albicans*, and 5.23% (11/210, CI 95% 3.79–6.67) with a codetection of both pathogens; however, the presence of infection did not correlate with any risk factors.

The stratification of patients indicated a significant difference in infection rate (*T. vaginalis*) according to age (*χ* ^2^ = 32.8, degrees  of  freedom = 6, *P* = 0.0001), as well as for *C. albicans* (*χ* ^2^ = 50.4, degrees  of  freedom = 6, *P* = 0.0001); however, prevalence rate was not significantly correlated with age (*r* ^2^ = 0.207, *P* = 0.97, by second-order polynomial regression) for *T. vaginalis* infection, as well as (*r* ^2^ = 0.234, *P* = 0.55 by second-order polynomial regression) for *C. albicans* infection; for both infections there was no association with marital status of participants (single versus married) (*P* = 0.729 for *T. vaginalis* infection, *P* = 0.466 for *C. albicans* infection, by Fisher's exact tests); there was no statistically significant association between the presence of infection with alcohol consumption (*P* = 0.761 for *T. vaginalis* infection, *P* = 1.000 for *C. albicans* infection, by Fisher's exact tests); when smoking was analyzed as a risk factor in this population, the results showed that the consumption of snuff (smoker, ex-smoker, and nonconsumer of snuff) had no association with the presence of infection for *T. vaginalis* (*χ* ^2^ = 0.906, degrees  of  freedom = 2, *P* = 0.636), as well as (*χ* ^2^ = 0.674, degrees  of  freedom = 2, *P* = 0.714) for *C. albicans* infection. Moreover, the presence of discomfort (burning, itching, and vaginal secretion) had no association with the presence of infections (*P* = 0.169 for *T. vaginalis* infection, *P* = 0.761 for *C. albicans* infection, by Fisher's exact tests). Finally, a significant association in the number of sexual partners (two versus more than two) was observed for *T. vaginalis* infection (*P* = 0.047, by Fisher's exact test) but not for *C. albicans* infection (*P* = 0.248, by Fisher's exact test).

## 4. Discussion

The diagnosis of *Candida *spp is difficult, existing up to 50% of asymptomatic cases, moreover, *Candida* spp, is considered endogenous flora normal of the vagina, in growth limited conditions [13]; on the other hand, the presence and trichomoniasis symptoms depend on local immunity and the amount of parasites inoculated, and transit may be asymptomatic in 50% of cases [14]. *T. vaginalis* and *C. albicans* are considered the pathogens found in a more frequent in vaginal infections. The culture is the gold standard test for diagnosis of *C. albicans* and *T. vaginalis* infections in cases of vaginitis [15]; however, PCR is currently used for diagnosis of *T. vaginalis* and *C. albicans* obtaining a sensitivity and specificity of 100% [16, 17].

As it is known, the frequency of cases of candidiasis and trichomoniasis varies according to the studied population [18]. In this study, we found a high prevalence rate in cases of trichomoniasis and candidiasis considering that the study was conducted in a low-risk population and that most women were homemakers, in relation to the data reported in Mexico in 2010 for cases of trichomoniasis and candidiasis [9], and compared with different work in different studied populations [19–22], even in high-risk populations such as sex workers [23]. Moreover, unlike other reports, this study found cases in all age groups, with a larger number of cases for *T. vaginalis* in people over 50 years old, and cases of candidiasis were observed in almost the same way in all groups with a slight increase in the group of 21–30 years.

Risk factors for acquisition, the clinical characteristics, and significance of candiduria have been published primarily in relation to intensive care unit and immunocompromised. *Diabetes mellitus*, prolonged use of antibacterial agents, indwelling urinary catheter, genitourinary tuberculosis, chronic renal failure, malignancy, neutropenia, immunosuppressive therapy, urinary tract instrumentation, surgery, renal graft, female sex, and extremes of age are known risk factors of acquisition of candiduria [24]. Moreover, the presence of *Candida* in urine may represent contamination of clinical sample, actual colonization of the lower urinary tract, or may be a true indicator of invasive infection of lower and/or upper urinary tract [25]; also, the epidemiology of *Candida* infections has changed over the last two decades. The number of patients suffering from such infections has increased dramatically and the *Candida* species involved have become more numerous [26]. 

A feature of this study is that the majority of participants reported no history of STIs submitted; furthermore, there was no relationship between the presence of positive case and the presence of symptoms, only in the case of coinfections (14%) where a significant association with the presence of pain was observed, these results indicated that the majority of individual infections with *T. vaginalis* and/or *C. albicans* remain in an asymptomatic form; presumably because pathogens can remain in low concentrations. These results strongly suggest that the symptoms not always must be a secure evidence of disease [27]. A limitation of this study was that most of the participants were women; only 16.6% of the population were males, a population where infection with *T. vaginalis* and generally any STI has been difficult to characterize [28]. But despite the low participation of the population, samples were positive for both pathogens, and in some cases in the presence of co-infections, these findings reinforced the absence of symptoms plus the presence of asymptomatic cases of this type of infection. Perhaps the lack of laboratory techniques for an accurate diagnosis, as well as early confirmation, is what affects the permanence of the infection as well as the likelihood of having any of the serious and important consequences caused by these pathogens.

The sociocultural and educational levels are crucial in sexual behavior and the risk of STIs, if you consider this aspect to be able to identify risk factors for use in these populations in intervention programs to change the sexual behavior of these people [29]. However, in this studied population not determining factors for the presence of infections was present. In addition, there is some degree of promiscuity not clearly detected in the survey because the partner or husband could not be interviewed; however, possessing more than one partner was significant in the presence of infection as previously reported [30].

One of the most common associations with *T. vaginalis* is the presence of bacteria and *Candida* spp [31], and the data obtained in the group of pregnant women showed no association with the presence of infection risk factors analyzed (pain, history of STIs, itching and vaginal secretion, smoking, alcohol, etc.), but the association was observed with *C. albicans*. It is known that environmental changes such as increased glycogen production during pregnancy and altered levels of estrogen and progesterone, by the use of oral contraceptives, allow adherence of *C. albicans* to vaginal epithelial cells and facilitate germination of yeast [32]. These changes can transform asymptomatic colonization in symptomatic infection. Patients with changes in the level of estrogens and progesterone, as well as raising the pH and glycogen, can cause the growth and virulence of *T. vaginalis* [32, 33]. Hormone changes produced during pregnancy predispose a higher incidence of infections of the lower genital tract. This leads to maternal and perinatological complications. The diagnosis of *T. vaginalis* infection during pregnancy is of great importance as such infections are related to premature rupture of membranes, preterm delivery, and low birth weight [34].

## 5. Conclusion

In conclusion, the codetection of *T. vaginalis* and *C. albicans* by PCR in urine samples in a low-risk population attended in a clinic first level in central Veracruz, Mexico, is of great importance because the diversity of results observed in this study enrich the evidence that the etiology of this type of infection is variable and requires the individual studies to know the characteristics of the population with which they are working.

## Figures and Tables

**Table 1:** Socioeconomic characteristics and sexual behavior of the studied population.

	Age groups (years)															
	14–20		21–30		31–40		41–50		51–60		61–70		>71		Total	
	*N*	(%)	N	(%)	N	(%)	N	(%)	N	(%)	N	(%)	N	(%)	N	(%)
Schooling																
No study	0	—	2	0.793	2	0.793	6	2.380	7	2.574	8	2.941	7	2.778	32	12.698
Elementary	1	0.397	9	3.571	12	4.761	13	5.159	18	6.618	24	8.823	10	3.968	87	34.524
Secondary	25	9.921	11	4.365	11	4.365	10	3.968	8	2.941	5	1.838	0	—	70	27.778
High school	17	6.746	17	6.746	6	2.380	4	1.587	6	2.206	0	—	1	0.397	51	20.238
University	1	0.397	6	2.381	3	1.190	1	0.397	0	—	0	—	0	—	11	4.365
Postgraduate	0	—	1	0.397	0	—	0	—	0	—	0	—	0	—	1	0.397
Total	**44**	**17.460**	**46**	**18.253**	**34**	**13.492**	**34**	**13.492**	**39**	**15.476**	**37**	**14.682**	**18**	**7.142**	**252**	**100**
Marital status																
Single	20	7.936	10	3.968	11	4.365	13	5.158	11	4.365	14	5.556	6	2.380	85	33.730
Married	8	3.174	17	6.746	15	5.952	11	4.365	23	9.126	11	4.365	4	1.587	89	35.317
Other	16	6.349	19	7.539	8	3.174	10	3.968	5	1.984	12	4.761	8	3.174	78	30.952
Total	**44**	**17.460**	**46**	**18.253**	**34**	**13.492**	**34**	**13.492**	**39**	**15.476**	**37**	**14.682**	**18**	**7.142**	**252**	**100**
History of STIs																
Yes	1	0.396	1	0.396	1	0.396	1	0.396	4	1.587	0	—	1	0.396	9	3.571
No	43	17.063	45	17.857	33	13.095	33	13.095	35	13.888	37	14.682	17	6.746	243	96.428
Total	**44**	**17.460**	**46**	**18.253**	**34**	**13.492**	**34**	**13.492**	**39**	**15.476**	**37**	**14.682**	**18**	**7.142**	**252**	**100**
Smoking																
Smoker	0	—	3	1.190	1	0.396	3	1.190	1	0.396	0	—	0	—	8	3.174
Ex-smoker	6	2.380	6	2.380	1	0.396	4	1.587	5	1.984	7	2.777	7	2.777	36	14.285
Nonsmoker	37	14.682	38	15.079	32	12.698	30	11.904	31	12.301	29	11.507	11	4.365	208	82.539
Total	**44**	**17.460**	**46**	**18.253**	**34**	**13.492**	**34**	**13.492**	**39**	**15.476**	**37**	**14.682**	**18**	**7.142**	**252**	**100**
Consumption of alcohol																
Yes	21	8.333	21	8.333	12	4.761	13	5.158	12	4.761	14	5.556	6	2.380	99	39.285
No	23	9.126	25	9.920	22	8.730	21	8.333	27	10.714	23	9.126	12	4.761	153	60.714
Total	**44**	**17.460**	**46**	**18.253**	**34**	**13.492**	**34**	**13.492**	**39**	**15.476**	**37**	**14.682**	**18**	**7.142**	**252**	**100**
Sexual partners																
One	20	7.936	23	9.126	18	7.142	14	5.556	23	9.126	21	8.333	8	3.174	127	50.396
Two	11	4.365	14	5.556	4	1.587	9	3.571	6	2.380	7	2.777	3	1.190	54	21.428
More than two	7	2.777	8	3.174	9	3.571	10	3.968	8	3.174	9	3.571	7	2.777	58	23.015
None	1	0.397	0	—	1	0.397	0	—	0	—	0	—	0	—	2	0.793
Total	**44**	**17.460**	**46**	**18.253**	**34**	**13.492**	**34**	**13.492**	**39**	**15.476**	**37**	**14.682**	**18**	**7.142**	**252**	**100**
Condom use																
Yes	5	1.984	4	1.587	3	1.190	1	0.397	1	0.397	0	—	0	—	14	5.556
No	39	15.476	42	16.666	31	12.302	33	13.095	38	15.079	37	14.683	18	7.143	238	94.444
Total	**44**	**17.460**	**46**	**18.253**	**34**	**13.492**	**34**	**13.492**	**39**	**15.476**	**37**	**14.682**	**18**	**7.142**	**252**	**100**

*N*: samples.

**Table 2:** Age groups and positivity to *T. vaginalis* and *C. albicans* by PCR in urine samples.

Age (years)	Samples		Positive *T. vaginalis *		Negative *T. vaginalis *		Positive *C. albicans *		Negative *C. albicans *		Double positive	
	*N*	%	*N*	%	*N*	%	*N*	%	*N*	%	*N*	%
14–20	44	17.460	8	3.175	36	14.286	21	8.333	23	9.127	7	2.778
21–30	46	18.254	11	4.365	35	13.889	12	4.762	34	13.492	5	1.984
31–40	34	13.492	5	1.984	29	11.509	11	4.365	23	9.127	2	0.794
41–50	34	13.492	6	2.381	28	11.111	19	7.540	15	5.952	4	1.587
51–60	39	15.476	17	6.746	22	8.730	18	7.143	21	8.333	11	4.365
61–70	37	14.683	11	4.365	26	10.317	13	5.159	24	9.524	7	2.778
>71	18	7.143	1	0.397	17	6.746	4	1.587	14	5.556	0	—

Total	252	100	59	23.413	193	76.587	98	38.889	154	61.111	36	14.286

*N*: samples.

## References

[1] van Dam CJ (1995). HIV, STD and their current impact on reproductive health: the need for control of sexually transmitted diseases. *International Journal of Gynecology and Obstetrics*.

[2] OMS (2006). *Global Strategy for the Preventions and Control of Sexually Transmitted Infections*.

[3] WHO (2011). *First WHO Report on Neglected Tropical Diseases: Working to Overcome the Global Impact of Neglected Tropical Diseases*.

[4] Sobngwi-Tambekou J, Taljaard D, Nieuwoudt M, Lissouba P, Puren A, Auvert B (2009). Male circumcision and *Neisseria gonorrhoeae*, *Chlamydia trachomatis* and *Trichomonas vaginalis*: observations after a randomised controlled trial for HIV prevention. *Sexually Transmitted Infections*.

[5] Vecchiarelli A, Pericolini E, Gabrielli E, Pietrella D (2012). New approaches in the development of a vaccine for mucosal candidiasis: progress and challenges. *Frontiers in Microbiology*.

[6] Chaim W, Mazor M, Sobel JD (1997). *Candida albicans* vulvovaginitis-trends in care and implications. *Harefuah*.

[7] Mahmoudi Rad M, Zafarghandi A, Amel Zabihi M, Tavallaee M, Mirdamadi Y (2009). Identification of Candida species associated with vulvovaginal candidiasis by multiplex PCR. *Infectious Diseases in Obstetrics and Gynecology*.

[8] Helms DJ, Mosure DJ, Metcalf CA (2008). Risk factors for prevalent and incident *Trichomonas vaginalis* among women attending three sexually transmitted disease clinics. *Sexually Transmitted Diseases*.

[9] SINAVE/DGE/SALUD/Información Epidemiológica de morbilidad.

[10] Shen AL, Porter TD, Wilson TE, Kasper CB (1989). Structural analysis of the FMN binding domain of NADPH-cytochrome P-450 oxidoreductase by site-directed mutagenesis. *Journal of Biological Chemistry*.

[11] Kengne P, Veas F, Vidal N, Rey JL, Cuny G (1994). *Trichomonas vaginalis*: repeated DNA target for highly sensitive and specific polymerase chain reaction diagnosis. *Cellular and Molecular Biology*.

[12] Baquero C, Montero M, Sentandreu R, Valentin E (2002). Identification of *Candida albicans* by polymerase chain reaction amplification of a CaYST1 gene intron fragment. *Revista Iberoamericana de Micologia*.

[13] Ziarrusta BG (2002). Vulvovaginitis candidiásica. *Revista Iberoamericana de Micología*.

[14] Plourd DM (1997). Practical guide to diagnosing and treating vaginitis. *Medscape Women's Health*.

[15] Boeke AJP, Dekker JH, Peerbooms PGH (1993). A comparison of yield from cervix versus vagina for culturing *Candida albicans* and *Trichomonas vaginalis*. *Genitourinary Medicine*.

[16] Devillard E, Burton JP, Reid G (2005). Complexity of vaginal microflora as analyzed by PCR denaturing gradient gel electrophoresis in a patient with recurrent bacterial vaginosis. *Infectious Disease in Obstetrics and Gynecology*.

[17] Schwebke JR, Burgess D (2004). Trichomoniasis. *Clinical Microbiology Reviews*.

[18] Flores-Paz R, García-Jiménez E, Arriaga-Alba M (2003). Etiología de la infección cérvico vaginal en pacientes del Hospital Juárez de México. *Salud Publica de Mexico*.

[19] Matini M, Rezaie S, Mohebali M (2012). Prevalence of *Trichomonas vaginalis* infection in Hamadan City, Western Iran. *Iranian Journal of Parasitology*.

[20] Ginocchio CC, Smith JS, Aslanzadeh J, Snook J, Hill CS, Gaydos CA (2012). Prevalence of *Trichomonas vaginalis* and coinfection with *Chlamydia trachomatis* and *Neisseria gonorrhoeae* in the United States as determined by the Aptima *Trichomonas vaginalis* nucleic acid amplification assay. *Journal of Clinical Microbiology*.

[21] Piperaki E, Theodora M, Mendris M (2010). Prevalence of *Trichomonas vaginalis* infection in women attending a major gynaecological hospital in Greece: a cross-sectional study. *Journal of Clinical Pathology*.

[22] Pattullo L, Griffeth S, Ding L (2009). Stepwise diagnosis of *Trichomonas vaginalis* infection in adolescent women. *Journal of Clinical Microbiology*.

[23] Haseen F, Hossain ME, Huq M (2012). Sexually transmitted infections and sexual behaviour among youth clients of hotel-based female sex workers in Dhaka, Bangladesh. *International Journal of STD & AIDS*.

[24] Fraisse T, Crouzet J, Lachaud L (2011). Candiduria in those over 85 years old: a retrospective study of 73 patients. *Internal Medicine*.

[25] Singla N, Gulati N, Kaistha N, Chander J (2012). Candida colonization in urine samples of ICU patients: determination of etiology, antifungal susceptibility testing and evaluation of associated risk factors. *Mycopathologia*.

[26] Rodloff AC, Koch D, Schaumann R (2011). Epidemiology and antifungal resistance in invasive candidiasis. *European Journal of Medical Research*.

[27] Anderson MR, Karasz A (2005). How do clinicians manage vaginal complaints? An Internet survey. *Medscape General Medicine*.

[28] Munson KL, Napierala M, Munson E (2013). *Trichomonas vaginalis* male screening with transcription-mediated amplification in a community of high sexually-transmitted infection prevalence. *Journal of Clinical Microbiology*.

[29] Periago MR, Fescina R, Ramón-Pardo P (2004). Steps for preventing infectious diseases in women. *Emerging Infectious Diseases*.

[30] Canchihuaman FA, Carcamo CP, Garcia PJ (2010). Non-monogamy and risk of infection with *Chlamydia trachomatis* and *Trichomonas vaginalis* among young adults and their cohabiting partners in Peru. *Sexually transmitted infections*.

[31] Perazzi BE, Menghi CI, Coppolillo EF (2010). Prevalence and comparison of diagnostic methods for *Trichomonas vaginalis* infection in pregnant women in Argentina. *Korean Journal of Parasitology*.

[32] Secretaría de Salud (2008). *Guía de Práctica Clínica: Diagnóstico y Tratamiento de la Vaginosis Infecciosa en Mujeres en Edad Reproductiva, en el Primer Nivel de Atención*.

[33] Owen MK, Clenney TL (2004). Management of vaginitis. *American Family Physician*.

[34] Cotch MF, Pastorek JG, Nugent RP (1997). *Trichomonas vaginalis* associated with low birth weight and preterm delivery. *Sexually Transmitted Diseases*.

